# Mrs. Eddy

**Published:** 1902-02

**Authors:** 


### Mrs. Eddy.

Mrs. Mary Patterson Baker Glover Eddy—Frye?—head of the "Christian Science" church (*sic*), recently was scathingly arraigned in a public lecture at Boston by Attorney Frederick W. Peabody. He openly charged Mrs. Eddy with conspiring to kill one Daniel Spofford, pointing out that her then husband was Asa Eddy, who was indicted by a grand jury for conspiracy to murder Spofford. He further stated that at the present time Mr. C. A. Frye, who poses as Mrs. Eddy's servant, footman, secretary, and man-of-all-work, and who holds the title to all her real and personal property, including jewels, is really her husband, and that he is living with her as a husband. By giving him all her property, and she has assumed a tidy fortune out of her "religion," Mrs. Eddy has made herself "judgment proof." Mr. Peabody reiterates the statement that Mrs. Eddy is one of the greatest "religious" fakirs of all time. Yet she is believed in by thousands of good people who think she really received a personal communication from God. No other proof is needed to show how thin is the veneer of general intelligence even in this country of free education.—*Exchange.*

